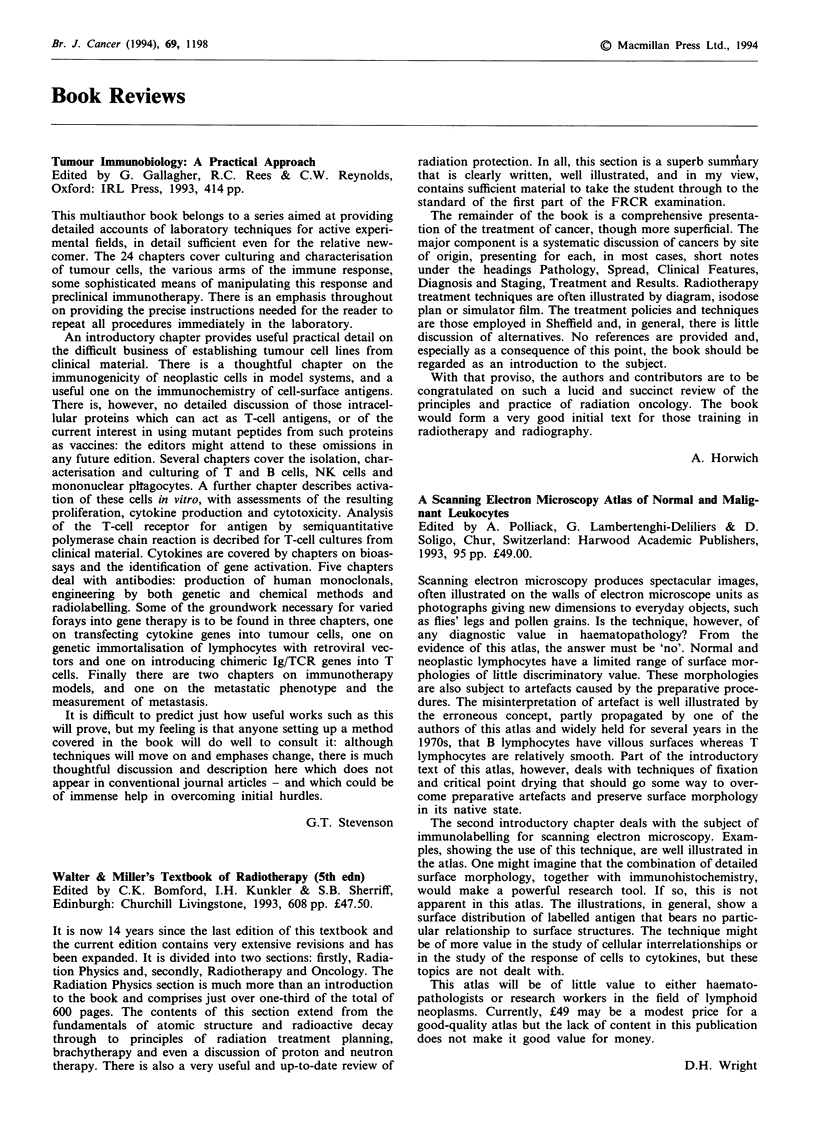# Walter & Miller's Textbook of Radiotherapy (5th edn)

**Published:** 1994-06

**Authors:** A. Horwich


**Walter & Miller's Textbook of Radiotherapy (5th edn)**
Edited by C.K. Bomford, I.H. Kunkler & S.B. Sherriff, Edinburgh: Churchill Livingstone, 1993, 608 pp. £47.50.

It is now 14 years since the last edition of this textbook and the current edition contains very extensive revisions and has been expanded. It is divided into two sections: firstly, Radiation Physics and, secondly, Radiotherapy and Oncology. The Radiation Physics section is much more than an introduction to the book and comprises just over one-third of the total of 600 pages. The contents of this section extend from the fundamentals of atomic structure and radioactive decay through to principles of radiation treatment planning, brachytherapy and even a discussion of proton and neutron therapy. There is also a very useful and up-to-date review of radiation protection. In all, this section is a superb summary that is clearly written, well illustrated, and in my view, contains sufficient material to take the student through to the standard of the first part of the FRCR examination.

The remainder of the book is a comprehensive presentation of the treatment of cancer, though more superficial. The major component is a systematic discussion of cancers by site of origin, presenting for each, in most cases, short notes under the headings Pathology, Spread, Clinical Features, Diagnosis and Staging, Treatment and Results. Radiotherapy treatment techniques are often illustrated by diagram, isodose plan or simulator film. The treatment policies and techniques are those employed in Sheffield and, in general, there is little discussion of alternatives. No references are provided and, especially as a consequence of this point, the book should be regarded as an introduction to the subject.

With that proviso, the authors and contributors are to be congratulated on such a lucid and succinct review of the principles and practice of radiation oncology. The book would form a very good initial text for those training in radiotherapy and radiography.

A. Horwich